# Welcome to *Neural Development*

**DOI:** 10.1186/1749-8104-1-1

**Published:** 2006-10-13

**Authors:** Andrew Lumsden, Bill Harris, Joshua R Sanes, Rachel Wong

**Affiliations:** 1 MRC Centre for Developmental Neurobiology, King's College London, Guy's Hospital, London SE1 1UL, UK; 2 Department of Physiology, Development and Neuroscience, Cambridge University, Downing St, Cambridge CB2 3DY, UK; 3 Department of Molecular and Cellular Biology and Center for Brain Science, Harvard University, 7 Divinity Ave, Cambridge MA 02138, USA; 4 Department of Biological Structure, University of Washington, HSB G514, Seattle, Washington 98195-7420, USA

Understanding how the nervous system develops poses one of the greatest challenges in biology. The nervous system is highly complex yet strikingly precise, both in its structural order and in its ability to perform the many functions required for survival and interaction with the environment. Such precision demands that key events at every developmental stage are executed properly and are coordinated to produce the circuitry underlying each of the adult nervous system's functions.

Over the years, the field of neurodevelopment has captured the creativity and curiosity of an increasingly large number of investigators. Diverse, state-of-the-art approaches have been used to study the developing nervous system of both vertebrates and invertebrates. These studies encompass investigations at all levels: understanding how neurons obtain their many and various identities, how they migrate to their appropriate locations, how they acquire their unique morphological and functional properties and, finally, how they form circuits that are appropriate. And the investigators – the developmental neuroscientists who will write, read and edit this journal – have a variety of reasons to be fascinated by these issues. Some see the nervous system as a fascinating (albeit not especially simple) model for addressing fundamental issues in developmental biology. Some, following the advice of Cajal, believe that mapping the establishment and plasticity of connectivity in the "young wood" of the embryo is a good way to find out what is going on in "the full-grown forest" of the adult. Still others are motivated by the growing realization that many neurological and behavioral disorders, even those manifested in adults, have their seeds in developmental defects. All will find common ground in the work we hope to highlight.

Understanding how the nervous system is built is best pursued by bringing together knowledge gained across many model systems; currently such findings are published in many journals, none of which is focused entirely on issues of neural development. The Editors of *Neural Development *thus believe that there is a need for a dedicated journal in this growing field, and given the rising popularity of open-access publication, we thought that a new open-access journal in our field is particularly timely.

Our idea behind *Neural Development *is to create a venue for publishing high-calibre work in all areas of nervous system development. We are dedicated to providing an outlet for full-length contributions that advance the field in any significant way. To ensure top-quality publication, we have enrolled a strong editorial board of international leaders in the field who will work hard to ensure that *Neural Development *becomes the cutting-edge journal and destination of choice for the best work in the field. We envisage that *Neural Development *will be an important platform for presenting the findings of those interested in the development of the nervous system at any and all levels.

Open access will allow key findings in our field to be more accessible and far-reaching, because it will be online and without charge. An online journal means more rapid reviews and shorter time to publication; for a growing field, dispensing new knowledge quickly is critical. The open access format will also allow us to publish articles without the constraint of page limitations. A fixed article-processing charge will be levied to cover the publication costs; there are no additional page or colour figure charges [1]. Furthermore, the dynamic nature of the developing nervous system is often best captured in the form of movies and animations. We have made this a priority in designing the format of *Neural Development*: movies will be built into the article, such that readers will be only one click away from a real-time view of the observations. Also, the online format permits the journal to generate a cover for every article we publish. Apart from the intellectual challenge of understanding how our favourite part of the nervous system develops, the sheer beauty of development is what has attracted many of us to the field.

We therefore provide the opportunity for authors of each article to highlight their work through their chosen cover image.

The Editors are committed to making this new venture a success for the neurodevelopment community. We hope that you share our vision and will join us in launching *Neural Development*.
